# Prospective Surveillance Screenings to Identify Physical Therapy Needs During Breast Cancer Diagnosis and Surviviorship: A Case Report

**DOI:** 10.7759/cureus.5265

**Published:** 2019-07-29

**Authors:** Cynthia Marsili, Christopher M Wilson, Nathan Gura

**Affiliations:** 1 Physical Therapy, Beaumont Health, Sterling Heights, USA; 2 Physical Therapy, Oakland University, Rochester, USA; 3 Physical Therapy, Ascension St. John Hospital Family Medical Center, St. Clair Shores, USA

**Keywords:** shoulder, manual therapy, mastectomy, therapeutic exercises, oncology, rehabilitation, fatigue, pain, prospective surveillance

## Abstract

Breast cancer and its treatments can cause detrimental effects to function and quality of life (QoL). These patients do not conventionally receive physical therapy services until impairments and functional limitations have become extensive. Emerging treatment models advocate for early rehabilitation screenings and proactive interventions, which are termed prospective surveillance. The purpose of this case report was to describe two prospective surveillance screenings at initial diagnosis and survivorship and subsequent physical therapy episodes of care for a patient with breast cancer. A 39-year-old female was diagnosed with invasive ductal carcinoma of the right breast. Approximately three months after the initial diagnosis, the patient had a right nipple-sparing mastectomy and immediate reconstruction with an expander. In addition, one lymph node was removed and underwent a biopsy, which was negative for metastases. The patient was screened by a physical therapist after her initial cancer diagnosis at the breast multidisciplinary clinic. This was after her mastectomy with an expander; the therapist recommended an episode of outpatient physical therapy due to impairments in pain, fatigue, loss of range of motion, weakness, and limitations in performance of her activities of daily living. The patient was seen initially for five visits. She underwent her final reconstructive surgery one month after discharge from physical therapy. Six months after her final reconstructive surgery, she was screened by the same physical therapist in the cancer survivorship clinic. Once again, therapy was recommended due to pain as well as deficits to her range of motion, strength, and functional status. The second episode of care lasted 14 visits and the patient showed improvements in pain, range of motion, shoulder strength and gains in the patient-specific functional scale and upper extremity functional index. This case reflects the importance of prospective surveillance screenings to overall patient outcomes. This patient may not have otherwise received physical therapy and its associated benefits without the prospective screenings by the physical therapist.

## Introduction

While the incidence of breast cancer has mostly been stable, mortality rates have been decreasing leading to increased breast cancer survivorship [1]. Cancer survivors often face many impairments that negatively affect their quality of life (QoL), occupational status, and ability to participate in meaningful activities. Patients may experience these impairments for two main reasons; side effects from cancer itself, or from treatments such as chemotherapy, radiation, or surgery. Physical impairments that are common to breast cancer specifically include decreased upper extremity range of motion (ROM) and strength, upper extremity and/or breast lymphedema, pain, fatigue, and loss of sensation [2]. While these physical factors may contribute to decreased QoL among cancer survivors, QoL may also be impacted due to psychological and other psychosocial components along with the physical components [3]. This is important for health care practitioners as efforts to address QoL factors may be multidimensional.

While 53% of adult-onset cancer survivors report problems with physical function as a result of their cancer and/or treatment, it is estimated that only 2% of cancer survivors receive interventions for these issues [4-5]. There is still room for improvement to attain the vision of the Academy of Oncologic Physical Therapy of the American Physical Therapy Association which envisions “unencumbered access to physical therapy services for cancer patients and survivors” [6]. This pattern of under-referral may be due to a decreased awareness or discomfort about the roles of rehabilitation team members by medical providers [7]. The Institute of Medicine, recognizing the need for continued care for cancer survivors, wrote guidelines and recommendations which include interdisciplinary screens and monitoring of cancer survivors [7]. Stout and colleagues recommended elevating awareness among health care providers, patients, and payers about the need for thorough integration of rehabilitation to optimize quality care [8]. They also include recommendations for rehabilitation screens and assessments from diagnosis through recovery, and incorporating objective assessments of the patient’s functional status before and during cancer intervention, and during survivorship [8].

While many breast cancer survivors do not receive treatment for their physical impairments, whether due to under-referral or lack of screening, it is also noted that there is a dearth of information in the literature on how to best manage these impairments [2]. In particular, there is a need for updated clinical practice guidelines on assessment and treatment of lymphedema and upper extremity musculoskeletal impairments in breast cancer survivors [2]. The purpose of this case report was to describe two prospective surveillance screenings and subsequent physical therapy episodes of care for a patient treated for breast cancer. The first screening occurred at the multidisciplinary clinic following the tumor board. At this time the patient had a mastectomy with a tissue expander. The second screening was after the removal of the expander with a permanent breast implant during a survivorship multidisciplinary clinic visit. The patient provided written consent for this case report and the report was composed utilizing the CARE Guidelines for reporting case reports [9].

## Case presentation

Patient history

The patient was a 39-year-old female diagnosed with invasive ductal carcinoma of the right breast, grade 1 at two sites. She initially felt a palpable lump in her right breast with nipple deformation. Her past medical history was significant for gastroesophageal reflux disorder (GERD) and prior upper respiratory infections. She was married with two teenage daughters and had a very supportive family who would do almost all activities for her during her initial recovery. Her primary language was Arabic, but she understood and could speak some English. She was a homemaker and her hobbies included cooking. Via mammogram, the two initial tumors were well-differentiated and were 1.5 x 1.6 x 1.5-cm mass at 10:00 approximately 2 cm from the nipple, while the other mass was 1.0 x 1.3 x 1.2 cm at 10:00 approximately 8 cm from the nipple. There was minimal deformation of the nipple upon the diagnostic examination. The tissue histology demonstrated both tumors were estrogen receptor (ER) positive, progesterone receptor (PR) negative, human epithelial growth factor receptor (HER)2/neu negative. Her final diagnosis was pT2, N0, Mx. Approximately three months after the initial diagnosis, the patient had a right nipple-sparing mastectomy and immediate reconstruction with an expander. In addition, one lymph node was removed and underwent a biopsy, which was negative for metastasis.

One month after surgery, the patient was seen by the tumor board, which included a physician, nurse navigator, physical therapist, and the patient along with her family among others. One service this tumor board provided was a multidisciplinary clinic (MDC) which included a physical therapist screening, a nurse navigator consultation, and a social work consultation. As the sentinel lymph node was negative, no additional surgery, chemotherapy, or radiation was recommended. The patient was recommended for follow-up with the local breast cancer support group, the hospital’s patient cancer resource center, the hospital’s Women’s Urology Center, and integrative medicine. It was determined during the physical therapist’s screen that the patient would benefit from physical therapy (PT) due to a moderate limitation in her right shoulder range of motion, requiring minimal assistance with dressing, pain when reaching with the right upper extremity, and difficulty sleeping due to shoulder pain and stiffness. Due to the nature of the screening, specific objective measures were not completed except for upper extremity anthropometric measures, which demonstrated that the left and right arms were nearly equal, which indicated that lymphedema was not a primary issue.

Patient Information

**Figure 1:**
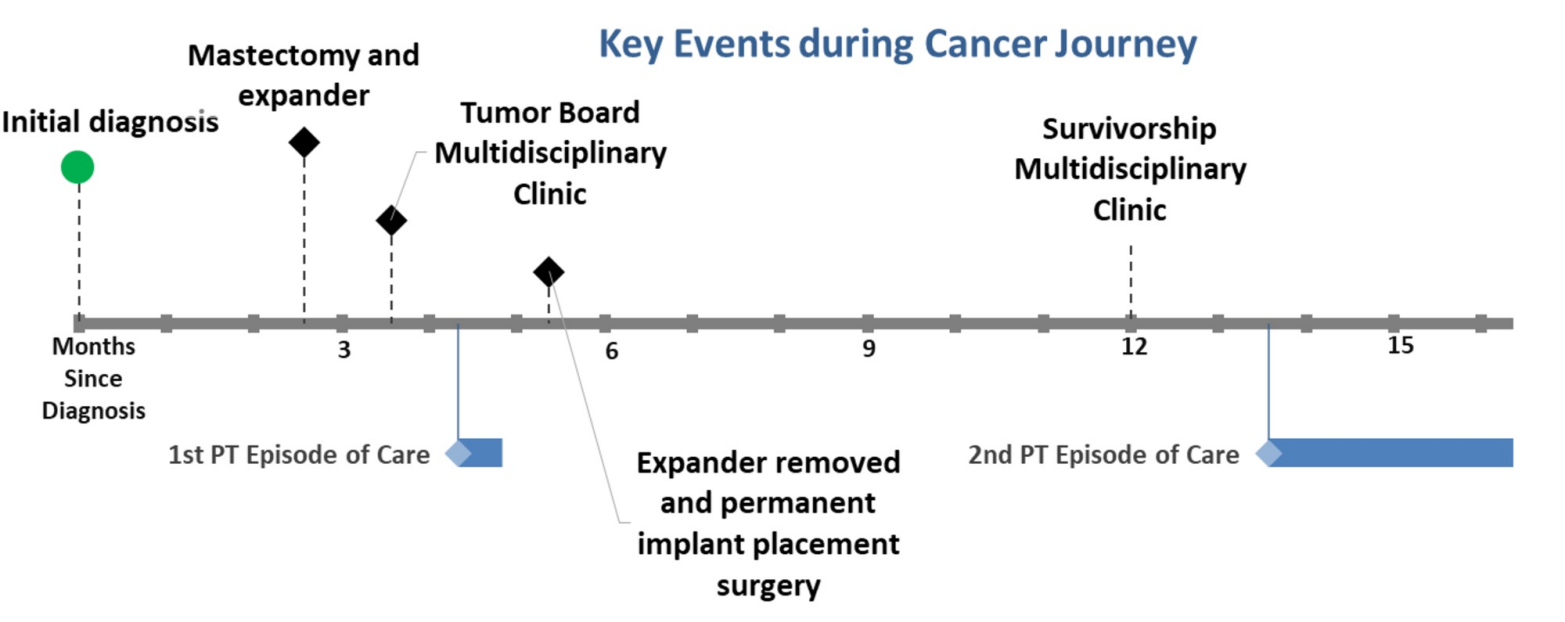
Timeline of key events during cancer journey Black diamond = surgical procedure or clinic visit; blue diamond/bar = physical therapy episode of treatment and duration; PT = physical therapy

During her cancer journey, she was screened twice by the same physical therapist. Each screening resulted in an episode of PT. See Figure 1 for the timeline of key events. After her initial diagnosis and surgery, she received a PT screen at the MDC which resulted in an episode of PT care. She also received another PT screen after her cancer treatment was completed in the cancer survivorship clinic. The patient reported that the only reason that she came to the survivorship clinic after her reconstructive surgery was because of the prior relationship between the therapist and patient. After the therapist recommended another episode of PT, the patient agreed and cited that her reason for receiving this episode of care was due to the trust built between patient and physical therapist.

First physical therapy episode of care 

The patient was initially seen for five PT visits that began approximately four and a half months after diagnosis to address impairments which included right upper extremity dysfunction, dizziness, muscle guarding, and low activity tolerance.

Examination

Subjective history: The patient presented with a diagnosis of invasive ductal carcinoma of the right breast stage 1. Due to the patient’s surgical history of a mastectomy with expanders, it was anticipated that she would have restrictions in upper extremity motion and she also reported chest tightness. She also reported substantial fatigue and pain (8/10 on the numerical pain rating scale), which are typical symptoms experienced by patients with cancer. The patient reported that she was receiving assistance from family members for her activities of daily living (ADLs) and had difficulty with grooming, dressing, and reaching.

Systems review: It was revealed that the patient had episodes of GERD and bronchial spasms with excessive coughing. The diagnoses of GERD and bronchial spasms affected the patient’s ability to lay supine which would affect her treatment regimen. She also had a minor risk of developing lymphedema in her right upper extremity due to the mastectomy and lymph node excision as well as her sedentary lifestyle. 

Tests and measures: On the initial examination, the patient presented with shoulder pain of 8/10, along with limitation in right shoulder ROM (Table 1). She had subjective complaints of right shoulder and anterior chest wall tightness. The patient also scored a 39 on the Upper Extremity Functional Index (UEFI), indicating right upper extremity dysfunction. The patient demonstrated decreased ability to complete ADLs such as washing her hair, reaching overhead with the right upper extremity, driving, and completing housework. The patient rated her ability to complete these activities with the patient-specific functional scale (PSFS), and her scores are shown in Table 2. The clinical findings during the initial presentation showed significant impairment and dysfunction on both her PSFS and UEFI. Range of motion is an important and reliable measure with a 10-degree difference believed to be clinically important in patients with breast cancer [10]. In the UEFI, a change of 9-10 points is the minimal clinically important difference (MCID) [11], while the PSFS requires a change of 2-3 points to be considered clinically significant [11]. The functional assessment of cancer therapy-general (FACT-G) is a self-reported outcome measure that helps measure health-related QoL. It consists of four sections: physical well-being, social well-being, emotional well-being, and functional well-being. It has shown acceptable reliability and validity in older adults with cancer [12]. The FACT-G MCID for each sub-scale was stated to be 3 points [12]. During the examination, it was theorized that since her family would complete her ADLs for her, this prevented her using her arm for her ADLs, thereby preventing the improvement of her physical impairments. The patient and family reported difficulty understanding how involved the surgery was and the subsequent pain and physical impairments. The patient understood the need for PT due to the education provided by the PT during the multidisciplinary clinic screening.

**Table 1:** Active range of motion and strength for first episode of care Manual muscle testing according to Kendall. 2+ = moves through partial range of motion against gravity; 3- = gradual release from test position against gravity

Direction of Motion	Active Range of Motion (degrees)	Strength (Manual Muscle Test)
Right Shoulder Flexion	108˚	2+
Right Shoulder Extension	35˚​​​​​​​	3-
Right Shoulder Abduction	81˚​​​​​​​	2+
Right Shoulder Internal Rotation	90˚​​​​​​​	Not tested
Right Shoulder External Rotation	35˚​​​​​​​	2+

**Table 2:** Patient-specific functional scale scores for first episode of care 0= unable to perform activity; 10 = able to perform at same level as before injury or problem

Activity	Score
Grooming Hair	2
Reaching Overhead	2
Driving	4
Housework	2

Evaluation and Plan of Care

The patient had pain, ROM and strength limitations, and fatigue that were causing her difficulty performing functional activities such as reaching into a cupboard, dressing, lifting, and washing hair. She had significant muscular guarding and initially could not comfortably lay supine. It was anticipated that she would make good gains in ROM and strength due to the lack of confounding comorbidities. These gains would improve her tolerance to activity and function. She demonstrated a good motivation to rely less on her family. Once the muscle tightness and muscle guarding were addressed, she would be more successful with her exercise regimen and become less dependent on her family. This would be assessed and monitored via the FACT-G, PSFS, and UEFI.

The patient had many positive prognostic factors, including her motivation, positive relationship with the therapist, and her family support. The negative prognostic factors included her pain level, language barrier, anxiety about her diagnosis, and possible excessive assistance from family. Finally, Arabic was her primary language which required additional efforts via thorough explanations of her interventions and pictures for her exercises.

The episode of care was anticipated to last approximately four to six weeks with a frequency of two to three times per week. The planned interventions included myofascial release, passive range of motion, active-assisted range of motion, active range of motion, scar management and wound care, soft tissue mobilization, stretching, body mechanics and postural training, strengthening exercises, and a home program that included self-care activity retraining.

The short term goals were planned to be met in five to seven visits, while the long term goals were planned to be met in 12-15 visits. These goals included decreasing pain levels, improving glenohumeral range of motion, strength, and functional outcome scores. It was also a long term goal to have her be independent with a home exercise program. To achieve these goals, the patient initially received a gentle passive range of motion to her right upper extremity, myofascial release, education, and training on proper posture and body mechanics, and a home exercise program to work on and maintain function.

Outcome

For this episode of care, she was seen for only five visits and she continued to have pain and bronchial spasms. She cancelled the rest of her visits due to other medical issues and having additional reconstructive surgery; therefore, a thorough reassessment was not possible. She continued to have difficulty lying supine and would continue to have coughing spells. She still complained of fatigue and difficulty performing all of her exercises. Functional scores on the FACT-G, PSFS, and UEFI were not able to be reassessed due to the abrupt discharge. Subjectively it was noted that ROM and strength were progressing which may have reflected decreased pain and functional gains. However, this could not be objectively documented.

Second episode of care 

The patient underwent a final reconstructive surgery to remove her chest expanders approximately five months and 10 days after her initial diagnosis. After this, her cancer treatments had been concluded or stabilized, and the patient was seen at the survivorship clinic approximately six and a half months later. The survivorship clinic provided screens from a physical therapist, social worker, nurse navigator, and dietician. The PT screen found extreme fatigue, low activity tolerance, and right scapular and shoulder pain and tightness. Upper-extremity anthropometric measures were nearly equal, indicating that lymphedema was not an issue for the patient at this point. She subjectively reported a pain score of 6/10 and a fatigue score of 8/10. She also demonstrated a minimal limitation to her right upper extremity ROM. Due to this, the patient was recommended for outpatient PT services and was seen for an initial evaluation six weeks later. It was recommended that the patient initiate PT earlier but was unable to do so because of personal scheduling challenges.

Examination

**Figure 2:**
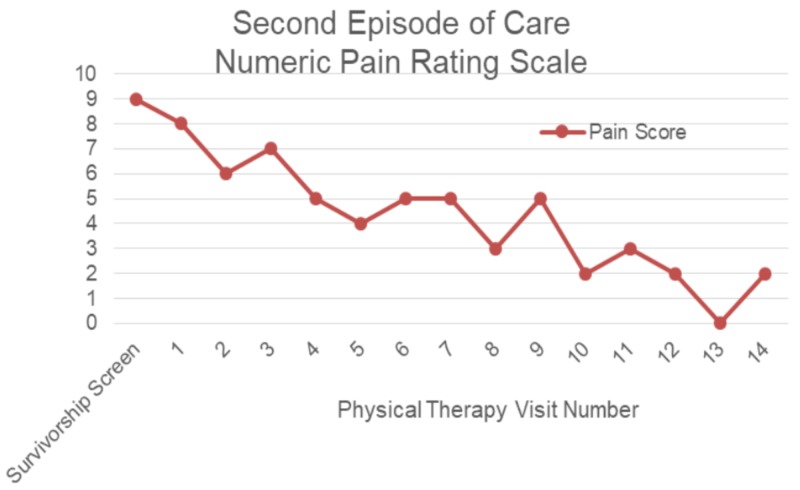
Pain rating for second episode of care 0 = no pain; 10 = worst possible pain

**Figure 3:**
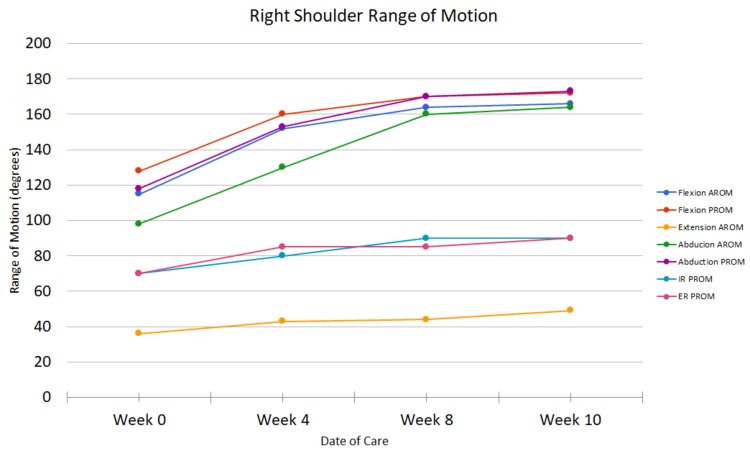
Shoulder range of motion for second episode of care AROM = active range of motion; PROM = passive range of motion; IR = internal rotation; ER = external rotation

**Figure 4:**
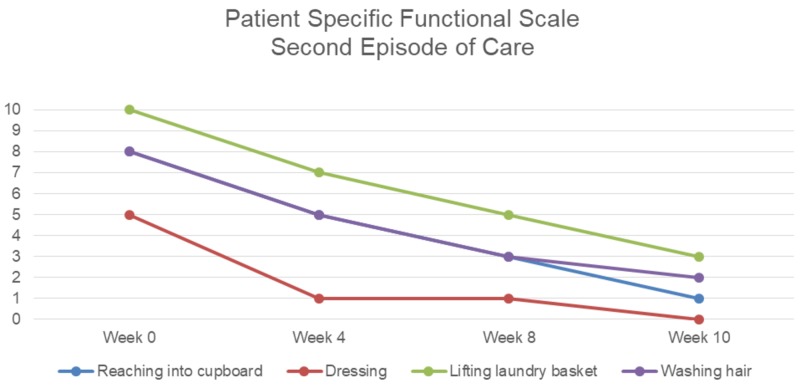
Patient-specific functional scale ratings for second episode of care 0 = least functional impairment; 10 = most functional impairment

**Figure 5:**
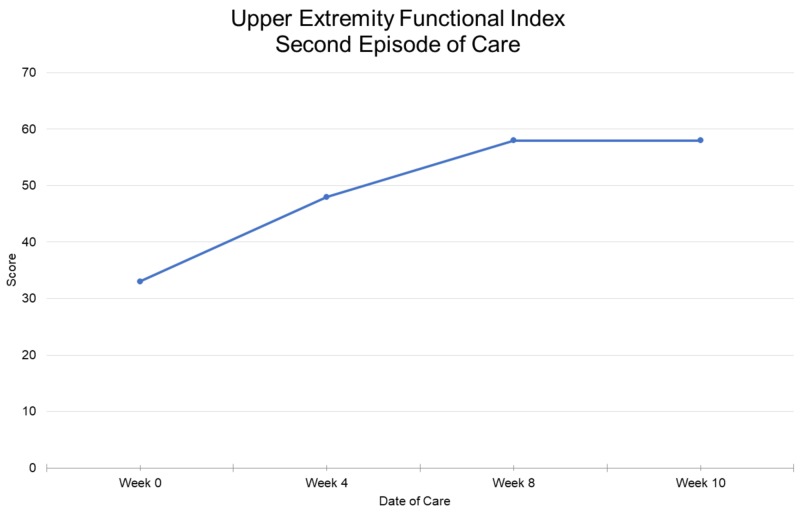
Upper extremity functional index for second episode of care 0 = most impaired function; 80 = least impaired function

Initially, when the patient was evaluated, she noted that she was sleeping excessively during the day due to fatigue and that she had to have her daughters assist her with ADLs such as showering. She reported anxiety with participation in PT and using her surgical arm. Due to muscle guarding and pain, it was anticipated that an initial focus on manual therapy would improve the patient’s tolerance to future exercises. She stated that she still had significant limitations in strength and ROM, while being able to do less functional reaching activities. The patient’s anxiety was more pronounced at this time due to her frustration of continuing to have pain and functional limitations. Her bronchial spasms and symptoms of GERD were under control at this point in her treatment. The patient had received her final reconstructive surgery eight months prior and did not receive any adjuvant cancer therapies. On the initial examination for the second episode of care, the patient’s pain scores, right shoulder range of motion, FACT-G, PSFS, and UEFI were taken. These measures were assessed regularly throughout treatment (Figures 2-5).

Evaluation and Plan of Care

Due to her mastectomy with expanders and reconstructive surgery, she did have limitations in mobility and pain that were contributing to her functional limitations. It was anticipated that the patient’s significant shoulder muscle tightness was due to overuse of her upper trapezius and levator scapulae musculature during upper extremity activities. The plan of care was to direct the patient in a safe exercise progression program, education on self-management techniques and manual therapy to address the objective impairments found in the examination. The goals were to improve shoulder range of motion, FACT-G, PSFS scores, UEFI scores, and for the patient to be independent with a home exercise program.

Intervention

The patient initially received education on proper posture and body mechanics, activity modification, myofascial release, soft tissue massage, ROM and strengthening exercises. She also was given a thorough home exercise program. Manual techniques were focused on decreasing the patient’s muscle guarding and pain, with the head of the bed initially elevated during treatment for her GERD. Gentle passive and active-assisted ROM was performed, as well as wall washing and supine external rotation stretches. The patient then progressed to active assisted shoulder exercises with a pulley and a cane, and then to resistive exercises with elastic exercise bands. At the end of her treatment, the patient received a thorough home exercise program (HEP) to maintain her functional gains outside of PT.

Based on the patient’s symptoms of muscle guarding, pain, and GERD, passive ROM of the patient’s right shoulder was done in a semi-supine position, which then was progressed to fully supine. This progressive positioning minimized the patient’s muscle guarding, pain, and eased the patient’s anxiety of moving her shoulder. The interventions focused on the impairments which then were translated to improved functional gains. In addition, education of the patient’s family about when to assist the patient was vital to the patient’s treatment. Thorough education on proper posture and body mechanics were performed. Because of a mild language barrier and being quite anxious due to having pain, her exercise program had to be progressed slowly and required extensive explanations. Her family never came to a session but it was discussed that she should rely less on them and be more active during the day. Improvements in ROM and pain resulted in an improved functional performance at home which subsequently reduced her anxiety.

Outcome

The second episode of care lasted 14 visits and the patient showed many improvements, including pain (Figure 2), range of motion (Figure 3), shoulder strength to an average of 4+ based on manual muscle testing, and PSFS scores (Figure 4). The patient also improved her UEFI score to a 58 and was independent with her HEP (Figure 5). The patient could lay flat in supine, had decreased muscle guarding, and used better mechanics when lifting her arm as noted by less excessive upper trapezius activation. Lee and colleagues reported that scapula-based exercise had beneficial effects on pain, QoL, and aspects of strength in a randomized controlled trial [13]. All of the interventions were aimed at improving tolerance to shoulder and scapular ROM to complete functional activities while keeping her pain low to ensure patient compliance and encouragement. The FACT-G demonstrated fluctuation throughout the treatment course. The total score did not demonstrate much significant change in from the beginning to end of treatment, which was not unexpected as QoL is multifactorial. There was a trend toward improvement in the physical and functional domains after the initial evaluation which may be the best measures of clinical efficacy for PT of the four FACT-G domains.

Follow-up and outcomes

When the patient first started PT, especially after reconstructive surgery, she was sleeping frequently throughout the day due to fatigue and had difficulty performing her ADLs. After her course of PT was completed, her anxiety decreased, she noted less reliance on her family and was even going out more with friends. Her ability to dress, reach, and drive all improved.

It was determined that the patient was ready for discharge based on her lower pain levels, ability to perform ADLs and independence with her HEP. During discharge, the importance of compliance with her HEP was emphasized to maintain her gains. Cessation of her HEP and allowing her family to perform her daily tasks and ADLs may have resulted in future dysfunction and disability. The patient also received instructions to notify her physician if her pain increased or her ROM became more restricted.

## Discussion

After each screening by the physical therapist (during initial MDC clinic after tumor board and the survivorship clinic screening), the patient demonstrated significant subjective, objective, functional and QoL issues that warranted PT. As a result of the physical therapist’s integration into these cancer services, the patient’s deficits were identified early and an expedited referral to PT was proactively initiated to address these impairments and functional limitations. 

The National Cancer Institute's recommendations for prospective screenings and integrated involvement of rehabilitation professionals do not consistently occur within the cancer population [8]. It is unclear as to why further integration has not occurred but it may be due to limited ability to dedicate a physical therapist to perform screenings. The screenings may be considered "non-productive" or "non-billable" time. A better metric of the effectiveness of physical therapist productivity or clinical effectiveness may be the screening-to-referral ratio which would demonstrate that appropriate referrals were initiated for a PT episode of care that might not have otherwise been identified or occurred.

As this case report described one patient with unique emotional, cultural, and psychosocial circumstances and treatment challenges, these results may not be generalizable to other patients with breast cancer. There were challenges in the management of this patient’s case due to multiple cancellations and other comorbidities which created a barrier to receiving PT. Throughout the patient’s treatments, the patient’s family was extremely helpful and did nearly everything for the patient which initially resulted in the patient not actively engaging in those activities and sleeping throughout the day. This may have limited the patient’s speed of recovery in the early stages of rehabilitation. This case is unique as there were multiple points of contact across the patient’s cancer journey, which allowed for early identification of the patient’s issues which may have resulted in a shorter and more efficient treatment course.

## Conclusions

This patient experienced a variable course of recovery after her breast cancer treatments. At initial diagnosis and her survivorship care plan visit, the patient was prospectively screened by a physical therapist. Both screens identified clinical needs that required formalized therapeutic interventions. After her reconstructive surgery, this patient verbalized the only reason she came to the survivorship program screening was because she wanted to be examined by the therapist with which she had a previous relationship. She reported that she was only amenable to PT for the second episode of care because of the previously established therapist-patient relationship. After discharge, she reported improvements in QoL, relied less on family, and even discussed seeking out part time employment. This case emphasizes the critical role of the physical therapist in prospective screenings throughout the continuum of care. If this patient had not had these screenings, her QoL issues may have remained unresolved.
